# Supplementary material for: Highly Efficient Synthetic CRISPR RNA/Cas9-Based Mutagenesis for Rapid Cardiovascular Phenotypic Screening in F0 Zebrafish
Source: Front Cell Dev Biol. 2021 Oct 22;9:735598. doi: 10.3389/fcell.2021.735598 (PMC8570140; doi:10.3389/fcell.2021.735598)

Supplementary Figure 1

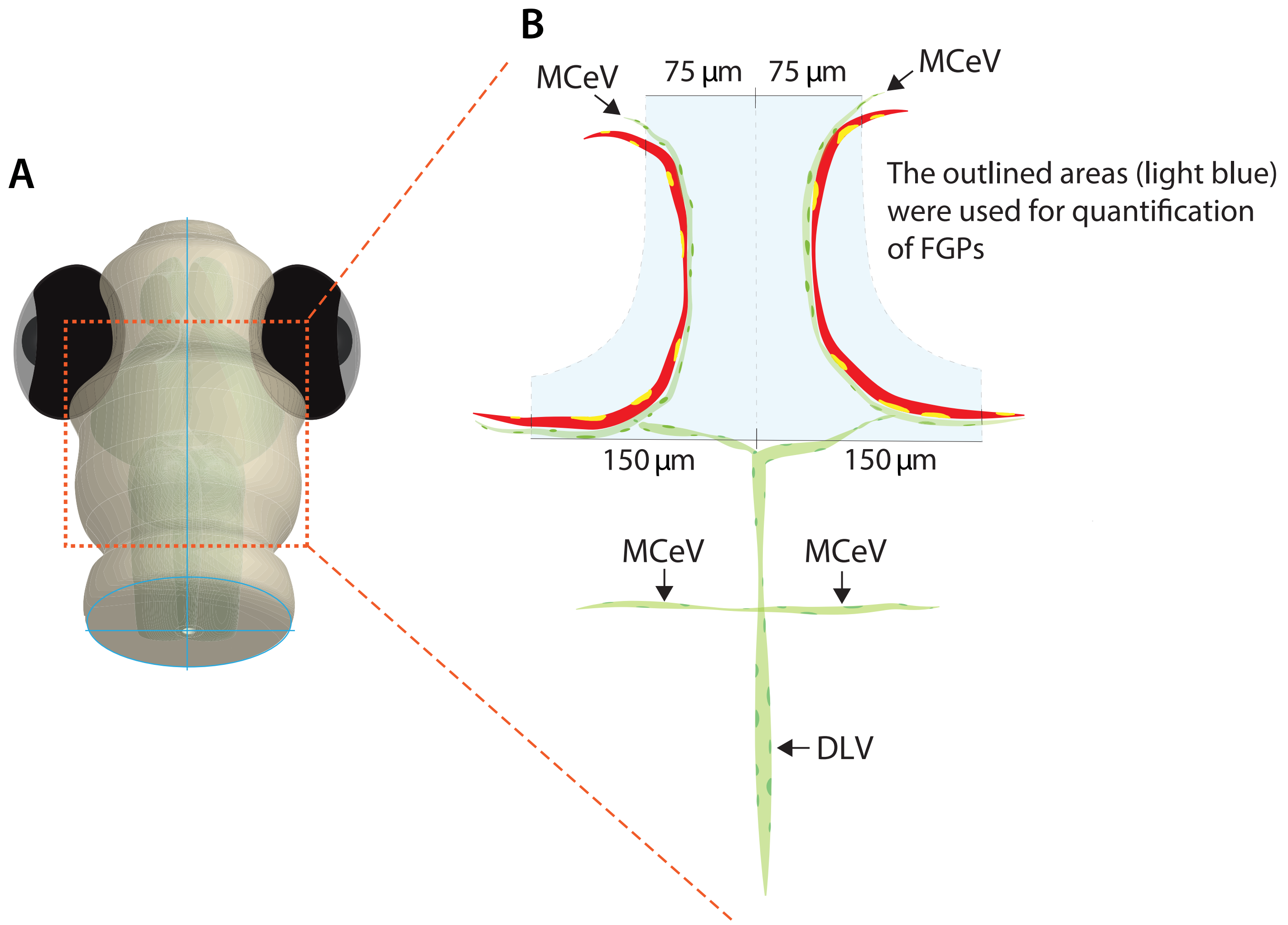

Supplementary Figure 2

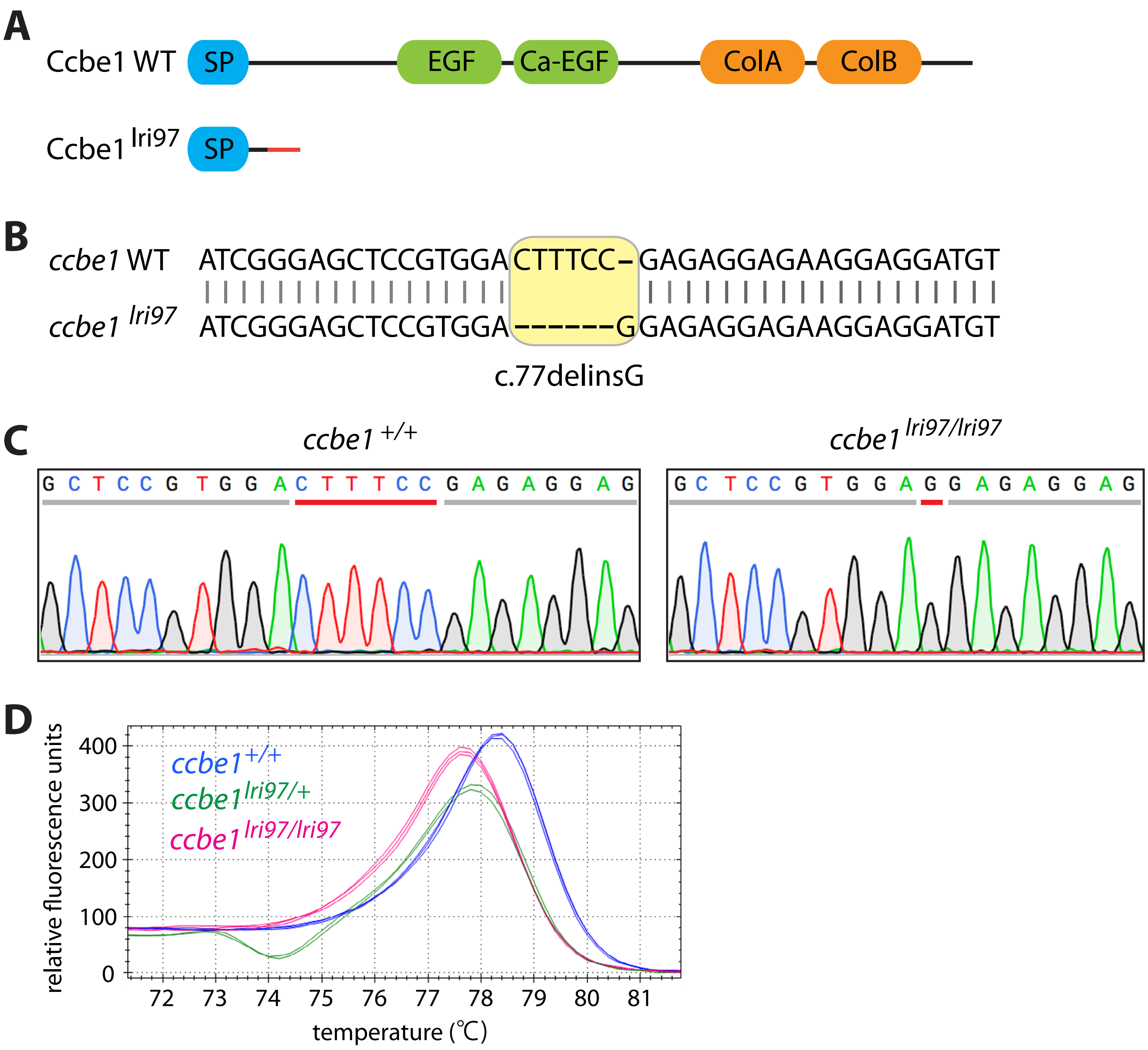

Supplementary Figure 3

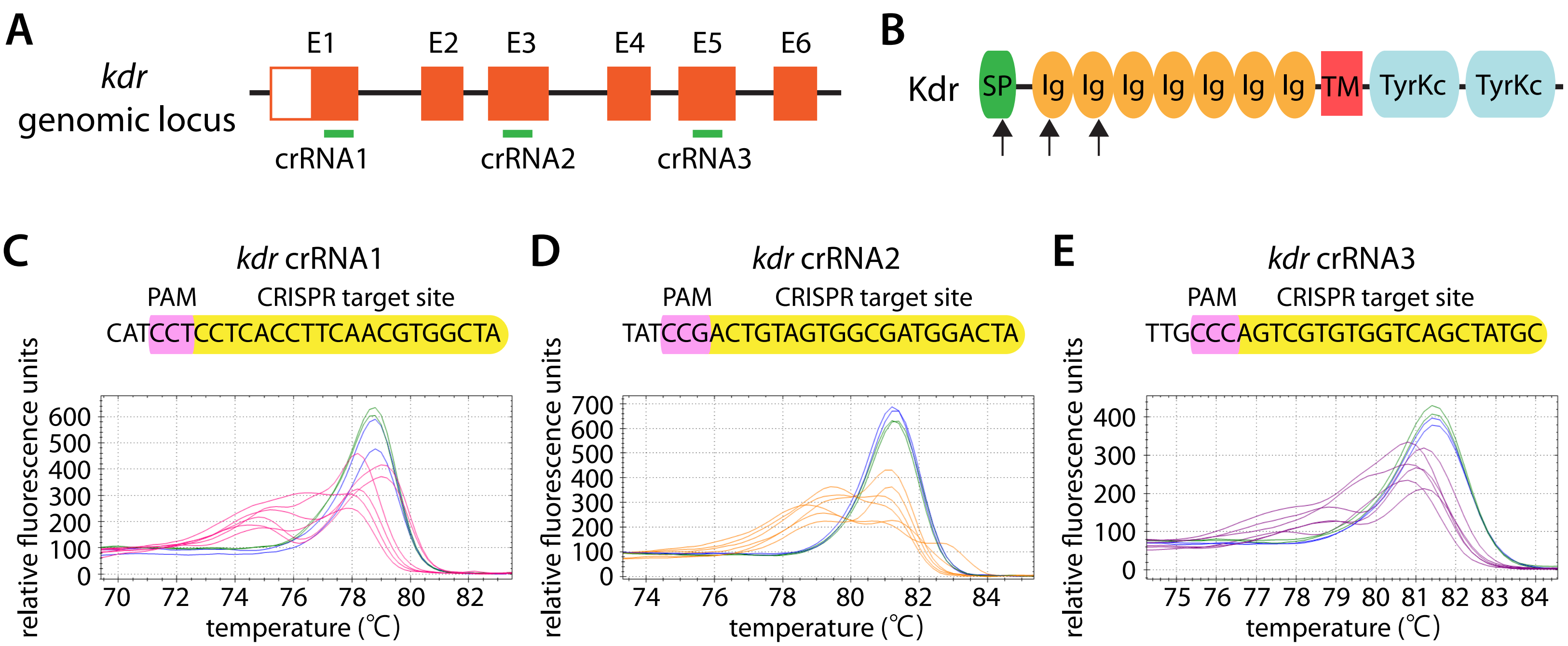

Supplementary Figure 4

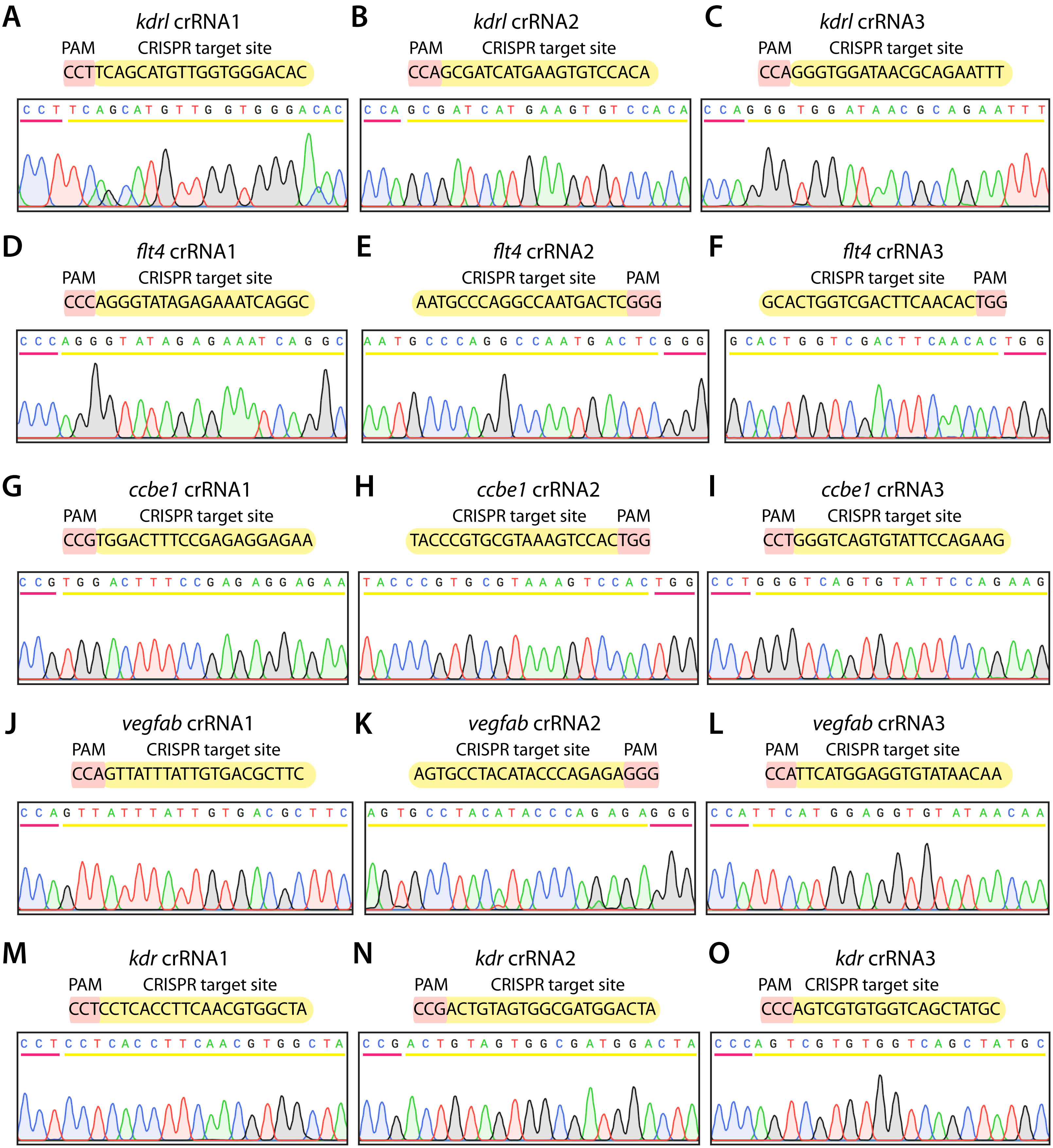

Supplement: Supplementary Figure 1 — Quantification of FGPs over the optic tectum. (A) Schematic representation of the dorsal view of the zebrafish larval head. The boxed area indicates the approximate location where the confocal images of bilateral Tg(lyve1:DsRed)+ loops over the optic tectum were captured. (B) Schematic diagram of the vasculature (green) and bilateral Tg(lyve1:DsRed)+ loops (red) over the optic tectum. The areas in which FGPs were quantified are highlighted in light blue. The number of Tg(fli1:nEGFP);Tg(lyve1:DsRed)-double positive FGPs (yellow) within the highlighted area were recorded. [file Image_1.pdf]
